# Clonal Expansion within Pneumococcal Serotype 6C after Use of Seven-Valent Vaccine

**DOI:** 10.1371/journal.pone.0064731

**Published:** 2013-05-28

**Authors:** Nicholas J. Loman, Rebecca A. Gladstone, Chrystala Constantinidou, Anna S. Tocheva, Johanna M. C. Jefferies, Saul N. Faust, Leigh O’Connor, Jacqueline Chan, Mark J. Pallen, Stuart C. Clarke

**Affiliations:** 1 Centre for Systems Biology, School of Biosciences, University of Birmingham, Birmingham, United Kingdom; 2 Molecular Microbiology Group, Sir Henry Wellcome Laboratories, Academic Unit of Clinical and Experimental Sciences, Faculty of Medicine, University of Southampton, Southampton, United Kingdom; 3 Division of Microbiology and Infection, Warwick Medical School, University of Warwick, Coventry, United Kingdom; 4 NIHR, Wellcome Trust Clinical Research Facility, University Hospital Southampton Foundation NHS Trust, Southampton, United Kingdom; 5 Health Protection Agency, Southampton, United Kingdom; 6 Southampton NIHR, Respiratory Biomedical Research Unit, University Hospital Southampton Foundation NHS Trust, Southampton, United Kingdom; Instituto Butantan, Brazil

## Abstract

*Streptococcus pneumoniae* causes invasive infections, primarily at the extremes of life. A seven-valent conjugate vaccine (PCV7) is used to protect against invasive pneumococcal disease in children. Within three years of PCV7 introduction, we observed a fourfold increase in serotype 6C carriage, predominantly due to a single clone. We determined the whole-genome sequences of nineteen *S. pneumoniae* serotype 6C isolates, from both carriage (n = 15) and disease (n = 4) states, to investigate the emergence of serotype 6C in our population, focusing on a single multi-locus sequence type (MLST) clonal complex 395 (CC395). A phylogenetic network was constructed to identify different lineages, followed by analysis of variability in gene sets and sequences. Serotype 6C isolates from this single geographical site fell into four broad phylogenetically distinct lineages. Variation was seen in the 6C capsular locus and in sequences of genes encoding surface proteins. The largest clonal complex was characterised by the presence of lantibiotic synthesis locus**.** In our population, the 6C capsular locus has been introduced into multiple lineages by independent capsular switching events. However, rapid clonal expansion has occurred within a single MLST clonal complex. Worryingly, plasticity exists within current and potential vaccine-associated loci, a consideration for future vaccine use, target selection and design.

## Introduction

*Streptococcus pneumoniae* (the pneumococcus) causes life-threatening invasive disease (pneumonia, septicaemia, and meningitis), primarily in children and the elderly, as well as other less severe infections (sinusitis, and acute otitis media). The global burden of pneumococcal invasive disease was estimated at almost 15 million cases in 2000.[1] Asymptomatic carriage is known to precede invasive disease and young children are the major reservoir of pneumococci in human populations, around one-third of children under five and over half of those under two carried *S. pneumoniae* asymptomatically in the nasopharynx.[2]–[4]

The pneumococcus is naturally transformable and shows considerable genotypic and phenotypic diversity, particularly in capsular serotype, of which over 90 are known. Capsular serotype is associated with the ability to cause invasive disease—around a quarter of the known serotypes account for the majority of invasive infections; some serotypes are at least ten-fold more likely to cause invasive disease than others.[5]

The seven-valent pneumococcal conjugate vaccine (PCV7) was licensed in the USA and Europe just over ten years ago. The vaccine consists of the polysaccharide capsule of seven serotypes associated with paediatric invasive disease in North America (4, 6B, 9V, 14, 18C, 19F and 23F), conjugated to diphtheria toxoid. It was added to the childhood immunisation schedule in the USA in 2001 but not added to the UK’s schedule until 2006. The use of PCV7 resulted in a reduction of invasive pneumococcal disease both in the North America and Europe.[6]–[9] However, since the introduction of PCV7, “serotype replacement” has been observed, with an increase in the proportion of invasive and non-invasive disease caused by serotypes not represented in the vaccine.[10], [11] In the UK, the increased incidence of invasive pneumococcal disease was caused by serotypes not included in the seven-valent vaccine to some extent offset the impact of PCV7.[9] For this reason, PCV7 has recently been replaced in UK and US vaccination schedules with PCV13, which provides coverage for six additional serotypes.

Serotype 6C was first described in 2007 as a subtype of 6A that differed in reactivity with monoclonal antibodies from the majority of 6A strains.[12] PCV7 contains polysaccharide from the 6B serotype, which provides protection against serotypes 6A and 6B.[13] However, such protection does not extend to serotype 6C.[14] Serotype 6C appears to be rare in pre-vaccination populations.[15]–[21] However, a worrying increase in the incidence of serotype 6C disease and carriage has been observed in diverse populations around the world since the introduction of PCV7.[22]–[32] Locally, since the introduction of PCV7, we have seen a fourfold increase in the proportion of nasopharyngeal serotype 6C isolates among pneumococci isolated from our study population in Southampton, England—from 3.8% of all isolates in the winter of 2006/7 to 13.5% and 13.7% in the winters of 2007/8 and 2008/9 respectively.[2], [33] Worryingly, we have also seen fatal cases of serotype 6C invasive disease.

Sustained surveillance and identification of factors influencing serotype distribution are essential for the continued control of pneumococcal disease and for rational vaccine design. Multi-locus sequence typing (MLST) has been used extensively to investigate the population structure of *S. pneumoniae*.[34] Although there is a correlation between MLST type and serotype, isolates from within a serotype can belong to a number of individual clonal complexes or sequence types and isolates of the same sequence type can express different capsular polysaccharides. Vaccine usage can result in capsular switching, where an existing sequence type from one capsular serotype remains prevalent by acquiring a different capsular locus.[35] Phylogenetic analyses of biosynthetic loci suggest that all 6C isolates belong to a single clade.[36] However, MLST studies on serotype 6C have shown it to encompass over two-dozen sequence types, several shared with the 6A serotype.[26], [27], [30], [36]

It is clear that pneumococcal strains from the same sequence type and/or serotype can differ significantly in gene content and virulence-associated phenotypes.[37] However, as MLST samples neutral sequence variation within a handful of housekeeping genes, it cannot always provide information about differences in gene repertoire or sequence variation within loci associated with virulence. Furthermore, MLST cannot discriminate between very closely related isolates. We therefore turned to a more informative approach: high-throughput whole-genome sequencing. Several recent studies have shown that this approach is capable of the ultimate resolution of a single nucleotide base-pair change (SNP) between isolates while also providing valuable information on differences in virulence loci and gene content.[38]–[41]

Recombination is the major driving force for short-term genome evolution in the pneumococcus—an early MLST study suggested that a single nucleotide site is approximately 50 times more likely to change through recombination than mutation, while a more recent whole-genome-sequencing study estimated that 88% of SNPs in the Pneumococcal Molecular Epidemiology Network clone1 (PMEN1) lineage were the result of recombination events.[41], [42] This high level of recombination distorts, but does not eliminate, phylogenetic signals of descent within pneumococcal lineages. However, given that recombination is so pervasive in pneumococci, evolutionary relationships between isolates are best represented by phylogenetic networks rather than by trees.[43]

### Study goals

We undertook whole-genome sequencing of multiple local isolates from serotype 6C to investigate genetic diversity within a single serotype in a constrained geographical location and time period, focusing on the sequence type driving current 6C expansion in our study population, ST1692. We used whole-genome sequencing of serotype 6C pneumococcal isolates from Southampton to address the following questions:

1. Can genome sequencing provide information comparable or superior to MLST on the evolution and spread of serotype 6C lineages within a single geographical location?
2. Are there differences in gene content among the serotype 6C strains circulating in our community and might these differences account for variation in the prevalence of different lineages?
3. Is there heterogeneity in capsule regions or other virulence factors that might influence virulence and impact on the development of future vaccines?

## Methods

### Bacterial isolates

Samples were collected during the winters of 2006-07, 2007-08 and 2008-09. To obtain pneumococcal carriage isolates, nasopharyngeal swabs were collected from children aged four years and under with written parental/guardian informed consent; ethical approval for this proceedure was obtained from Southampton and South West Hampshire Research Ethics Committee ‘B’ [REC 06/Q1704/105]. Samples were collected regardless of health and vaccination status, gender or ethnicity and no demographic data were collected. Children were swabbed only once. All serotype 6C invasive disease isolates (n = 4) from blood or cerebrospinal fluid specimens received by the HPA South East regional microbiology laboratory between 2006 and 2010 were included; these were all from adult patients. Microbiological procedures were performed as previously described.[2] Pneumococcal capsular typing was performed on genomic DNA isolated from sub-cultured isolates by multiplex PCR.[2] Multi-locus sequence type (MLST) was determined by Qiagen Genomic Services, using the MLST website www.mlst.net to assign sequence type (ST).[2], [34]

**Figure 1:**
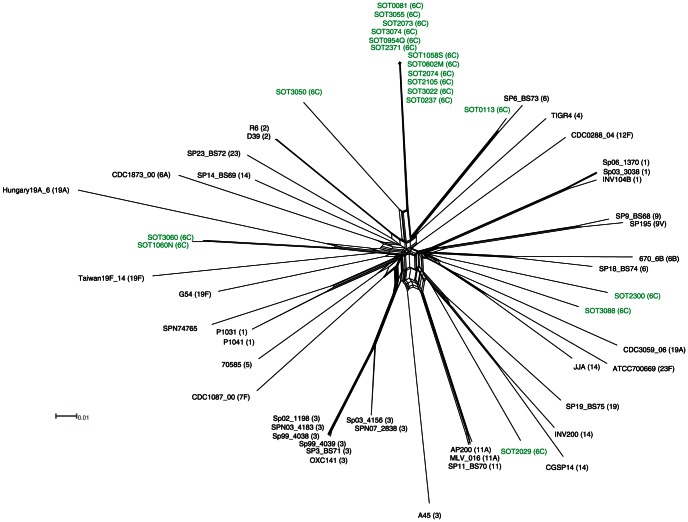
A phylogenetic split-network showing the relationship between Southampton 6C strains and other strains with whole-genome data from the public database. To generate the split-network, single nucleotide variants in concatenated multiple alignments of *S. pneumoniae* core genome coding sequences were input to a Neighbour-Net analysis in SplitsTree. Strains sequenced in this study are coloured in green. The serotype of each strain is shown in parenthesis.

**Figure 2:**
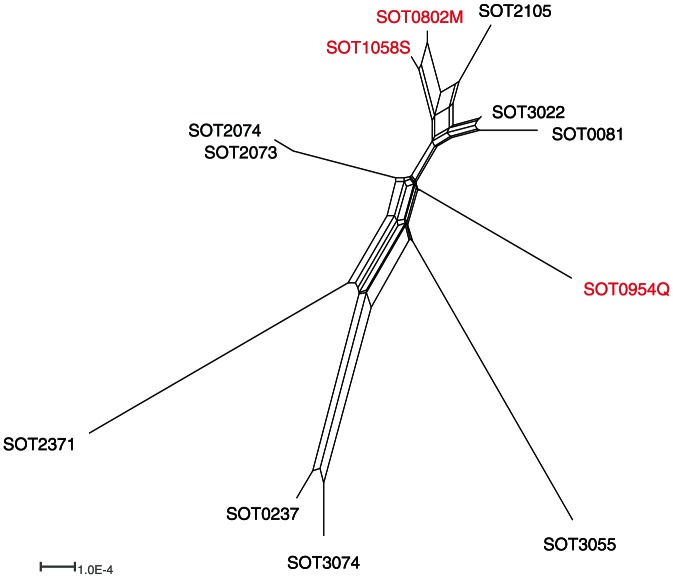
A phylogenetic split-network drawn from single nucleotide variants showing the relationship between CC395 isolates. Invasive isolates are coloured in red.

Thirty-two serotype 6C isolates were obtained from the carriage samples, falling into nine sequence types, of which ST1692 (belonging to clonal complex 395) was the most common (n = 18); three of the four invasive-disease isolates also belonged to ST 1692/CC395. CC395 was defined as all isolates within ST395 or within sequence types which shared 6/7 of MLST alleles with ST395. CC395 therefore encompassed nine isolates from ST1692, two representatives of ST1714 and a single representative of ST395. Fifteen of the 32 6C carriage isolates (47%) and four invasive disease isolates were selected for whole-genome sequencing (Table 1). These included at least one representative of each of the nine observed sequence types, with twelve isolates from CC395 (Table 1, Figure 1, 2).

**Table 1:** Pneumococcal isolates selected for whole genome sequencing.

Identifier	Year	Specimen type	Clinical Status and Outcome	ST
SOT0802M	2008	Blood	COPD exacerbation: Recovered	ST1692
SOT0954Q	2009	Blood	Pneumonia: Fatal	ST1692
SOT1058S	2010	CSF	Meningitis: Fatal	ST1692
SOT1060N	2010	Blood	Sepsis: Fatal	ST1150
SOT0081	2006/7	NP swab	Carriage	ST1692
SOT0113	2006/7	NP swab	Carriage	ST65
SOT0237	2006/7	NP swab	Carriage	ST1714
SOT2029	2007/8	NP swab	Carriage	ST3460
SOT2073	2007/8	NP swab	Carriage	ST1692
SOT2074	2007/8	NP swab	Carriage	ST1692
SOT2105	2007/8	NP swab	Carriage	ST1692
SOT2300	2007/8	NP swab	Carriage	ST1862
SOT2371	2007/8	NP swab	Carriage	ST395
SOT3022	2008/9	NP swab	Carriage	ST1692
SOT3050	2008/9	NP swab	Carriage	ST1600
SOT3055	2008/9	NP swab	Carriage	ST1692
SOT3060	2008/9	NP swab	Carriage	ST1150
SOT3074	2008/9	NP swab	Carriage	ST1714
SOT3088	2008/9	NP swab	Carriage	ST398

Abbreviations: CSF: cerebrospinal fluid; NP: nasopharyngeal.

### Procedures

DNA was extracted for whole-genome sequencing from an approximately 10^10^ bacterial cell pellet of obtained from six hours liquid culture in 10 ml of liquid culture Brain-Heart-Infusion (BHI) for whole-genome sequencing. Extraction was performed using the Qiagen 100/G genomic tips by following the manufacturer's instruction for Gram-positive bacteria. All strains were shotgun-sequenced by 454 (Roche, Welwyn Garden City) using Titanium reagents at the University of Birmingham sequencing service. The reference strain SOT2073 was also sequenced with 454 mate-pair sequencing with an insert size of 8 kb to produce a single-scaffold reference sequence. All sequences were submitted to the short-read archive (http://www.ncbi.nlm.nih.gov/sra) and are available under the accession number SRP013270.

An average of 40 million bp were generated per isolate, yielding approximately 19-fold coverage per genome. Mean read lengths were 331 bp. A *de novo* assembly was performed for each strain, using Newbler version 2.5 (Roche) under default settings except that the '-rip’ was invoked, which ensures that each read is placed in a single contig only. *De novo* assembly of each strain produced a mean of 167 contigs across all assemblies with a mean N50 (a statistical measure of the average length of a set of sequences) of 39753 base-pairs. Assembly of the strain SOT2073 data, including paired-end information, generated a single scaffold, 2,129,664bp in length, generally co-linear with the genome sequence of the well-characterised strain R6 (data not shown).

Assemblies were submitted to the xBASE annotation service (http://www.xbase.ac.uk/annotation) to provide an initial set of gene predictions. Orthologous genes were predicted from these annotated genomes in conjunction with complete *S. pneumoniae* genome sequences retrieved from GenBank using OrthoMCL (www.orthomcl.org) Whole-genome phylogenies were calculated from the core genome (defined as the set of single-copy orthologous genes present in all strains). Individual alignments of orthologues were produced by MUSCLE and trimmed for quality in Gblocks.[44], [45] The resulting alignments were concatenated into a single super-alignment and phylogenetic networks created using SplitsTree drawing on genomes of representative strains in the public databases. Additionally, whole-genome alignments were produced in Mauve and analysed with ClonalFrame to remove signals of recombination to provide phylogenetic signals of vertical descent. The datasets generated by this study, including assemblies, annotations, orthologue predictions and alignments have been deposited in a Github repository (http://github.com/nickloman/pneumococcus-6C-study).

For accurate read mapping of the capsule locus reads were mapped against the SOT2073 reference sequence using the gsMapper component of Newbler. SNP calls were also produced in this way. SNPs were filtered using a variant frequency cut-off of 100% and the effect on protein sequence determined using xBASE.

## Results

### Clonal diversity and clonal expansion

We identified SNPs in the genes conserved in all our isolates and other representative *S. pneumoniae* strains. From these, we generated a phylogenetic split-network (Figure 1), which largely reproduces the major pneumococcal lineages described by Donati *et al*.[43] Our serotype 6C isolates fall into four lineages. The largest 6C lineage consists of the twelve isolates belonging to clonal complex 395, together with SOT3050 from ST1600 and SOT0113 from ST65. Two serotype 6C isolates from ST1150—one associated with carriage (SOT3060), the other with fatal sepsis (SOT1060N) —form a distinct phylogenetic cluster. Two other seemingly unrelated serotype 6C carriage isolates, SOT2300 (ST1862) and SOT3088 (ST398) also form a distinct cluster. One carriage isolate from this serotype, SOT2029 from ST3460, sits separate from all other serotype 6C strains.

We next focused on diversity within this clonal complex, creating a phylogenetic network of CC395 strains (Figure 2). Tight clustering was found between four pairs of strains. The close relationship between SOT2073 and SOT2074 is unsurprising because these strains were collected from siblings in the same family during the same hospital visit. Nonetheless, there are seven SNPs that separate these genomes, three of them apparently acquired through recombination. Four of the seven SNPs occur in coding regions and three representing non-synonymous changes, emphasising the relatively fast pace of genome evolution in this species.

There are two pairs of carriage isolates (SOT0081/SOT3022 and SOT0237/SOT3074) where the isolates were obtained two years apart, yet cluster together tightly, suggesting that conserved genotypes can persist year on year. More worryingly, two of the four invasive isolates, SOT0802M and SOT1058S cluster together, yet were also obtained from samples two years apart, suggesting the persistence of a virulent clone carrying a serotype not covered by the PCV-7 vaccine.

### Sequence variation in loci associated with surface structures

Mapping alignments revealed that the number of SNPs separating each isolate from SOT2073 ranged from 8 to 19,829 among our serotype 6C isolates. Up to 128 SNPs were seen in isolates from ST1692 and up to 172 SNPs within the CC395 clonal complex (Table 2). Surprisingly, the majority of SNPs were non-synonymous, probably reflecting selective pressure on key virulence determinants. Known or putative virulence factors are commonly mutated including the IgA1 protease, choline-binding proteins, surface proteins PspA and PspC and endo-beta-N-acetylglucosaminidase. All isolates from CC395, together with isolate SOT3050 (ST1600), encode a second PspA-domain protein, not seen in any of the other sequence types. This protein contains two domains: a glucan domain with homology to PspA and a peptidase/caspase domain. The pair of invasive isolates, SOT0802M and SOT1058S, also show variation in the choline-binding protein PspC, in particular in the length of a proline-alanine repeat motif, linking the C-terminal choline-binding domain and the active peptide domain.

**Table 2:** Single nucleotide polymorphisms.

Strain	ST	Total SNP	Filtered	CDS	Non-Synonymous
SOT2074	1692	81	8	4	3
SOT2105	1692	217	67	49	30
SOT3022	1692	233	73	51	29
SOT802M	1692	202	100	77	45
SOT1058S	1692	237	109	77	46
SOT0081	1692	189	111	80	48
SOT954Q	1692	251	111	70	45
SOT3055	1692	304	128	102	70
SOT0237	1714	334	156	122	83
SOT3074	1714	353	161	123	82
SOT2371	395	367	172	123	81
SOT0113	65	16772	2466	2042	636
SOT2029	3460	18835	12148	10360	2977
SOT3050	1600	16534	13777	11551	3494
SOT3060	1150	17824	13892	11935	3466
SOT3088	398	17735	14506	12275	3721
SOT1060N	1150	19298	15832	13403	3874
SOT2300	1862	24959	19829	17209	5054

The number of filtered SNPs separating isolates from SOT2073 ranges from 8 to 19,829. Within ST1692 the largest number of SNPs is 304;within CC395, the largest number is 367.

The 6C capsular locus is thought to have originated at least three decades ago in a single recombination event that replaced the 6A *wciN* gene (encoding a glysoyltransferase) with the *wciN*-beta allele.[36], [46] However, we found heterogeneity in capsular gene sequences—in particular, several stretches from the capsular locus of isolate SOT0113 showed <95% sequence identity to the reference sequence from SOT2073 and other 6C isolates. Also, a previous described insertion in the *wzy* gene was found in SOT0113, but not in the other isolates examined.[47]

### Accessory genome of 6C serotype strains

Next, we examined the accessory genome of our serotype 6C isolates, that is the set of genes and gene clusters not present in all strains of *S. pneumoniae*. The largest differences in gene content between our 6C isolates and the SOT2073 reference genome were due to prophages, for example a near-identical ∼13 kb prophage in the two ST1714 isolates and the ∼32-kb *Streptococcus* phage 11865 in SOT3055. Prophages are known to carry virulence-related genes in many other bacterial species and, although a relatively underexplored topic, this is probably also true in the pneumococcus.[48], [49]

Our survey revealed several putative resistance genes within the 6C serotype. As noted, SOT2029 clusters separately from all other serotype 6C strains. Interestingly, this isolate, shows resistance to erythromycin and tetracycline but not penicillin (minimum inhibitory concentrations of >256, 6 and 0.064 respectively) and carries the *ermB* and *tetM* genes flanked by genes associated with conjugative transposons. All of the other isolates were sensitive to penicillin, tetracycline and erythromycin, and none contained sequences for *ermB* and *tetM* genes. All of our isolates from CC395 carry a lantibiotic synthesis locus not seen in any of the other serotype 6C sequence types we have studied. A very similar locus has been described in two other genome-sequenced *S. pneumoniae* isolates, CGSP14 and INV200.[50] However, these belong to a different serotype and are not related to CC395 in our phylogenetic network, suggesting that this cluster has undergone horizontal gene transfer.

## Discussion

This study illustrates the evolution of a single pneumococcal serotype, 6C, during a period of vaccine pressure. The scattered phylogenetic distribution of serotype 6C isolates provides convincing evidence for historical capsular serotype switching,[32] whereby the 6C capsular locus has been introduced into multiple pneumococcal lineages by independent recombination events. However, the phylogenetic network also confirms that, in our local population, the rise in prevalence of serotype 6C is largely due to clonal expansion within a single clonal complex.

The presence of a lantibiotic synthesis locus within and unique to all genome-sequenced members of this clonal complex provides a possible explanation for the success and clonal expansion of CC395 in our study group. Lantibiotics are bacteriocins that contain the modified amino acid lanthionine and which are produced by, and act on, Gram-positive bacteria.[51] Their ecological role is thought to impart colonisation resistance, preventing related strains from gaining a foothold in a specific environment [51], [52]. Our genomic survey suggests that lantibiotic production may have enhanced the fitness of CC395 in the presence of other *S. pneumoniae* lineages in a competitive and shifting environment, potenitally explaining its increasing predominance in our sample set.

As expected from previous studies, we found little or no variation in the complement or sequences of metabolic genes. Instead, we found a serotype 6C accessory genome largely composed of prophage genes. However, we did find further evidence of sequence variation within loci encoding surface structures, both proteins and capsular polysccahride, presumably driven by selective forces imposed by the host immune system. This is worrying for two reasons. Firstly, although the new vaccine PCV13 appears to provide protection against the 6C serotype,[53] our findings suggest that sufficient plasticity might exist within the 6C capsular locus for new vaccine-escape mutants to emerge; Elberse *et al.* [54] have made similar but distinct observations on plasticity within the 6C capsular locus. Secondly, given that surface proteins such as PspA and PspC are candidates for inclusion in next-generation protein–based pneumococcal vaccines, the sequence variation seen in these proteins raises the concern that the *S. pneumoniae* will respond to the introduction of these vaccines with the same kind of rapid evolution seen after use of polysaccharide vaccines. In other words, we may be facing an example of Red Queen evolutionary dynamics,[55] where we may need continual innovation in our vaccine repertoire to maintain the same level of control of infection. It is also worrying to see the emergence of antibiotic resistance in one of our 6C lineages.

This study confirms that genomic epidemiology surveys are no longer the sole preserve of the large sequencing centres and suggest that these techniques are poised to become cost-effective replacements for existing *S. pneumoniae* typing methods such as MLST, gene-specific PCR assays and PCR capsule typing. More generally, high-throughput sequencing provides a new tool in our armamentarium with the potential to keep pace with, or even outrun, the evolution and spread of microbial pathogens as the field developes further.
